# Syphilitic Dementia—Report of a Case

**Published:** 1902-05

**Authors:** Jas. B. Dillard

**Affiliations:** Davisboro, Ga.


### SYPHILITIC DEMENTIA—REPORT OF A CASE.

BY JAS. B. DILLARD, M.D.,

DAVISBORO, GA.

Strictly speaking, syphilitic dementia is not a typical definition for this case I am to report. A better definition, I think, would be a psycosis dependant upon syphilis. The infection may not at present exist making syphilis indirectly the cause of the brain trouble.

On the 28th day of August, last year, Mr. J., a merchant, called at my office for treatment with the following history: Age 63, a widower, tall and muscular, shoulders stooped, fair skin, blue eyes, family history good. He uses neither tobacco nor whiskey. Eighteen years prior patient was seized with a stroke of left facial paralysis, and the eye twitching is the only inconvenience he has experienced from the stroke.

The first part of the year 1896 patient contracted syphilis, and after exerting many efforts to perfect a cure he claimed he had utterly failed, and he was at this time suffering from the effects of the old case.

Edema of both lower limbs, with dark purplish spots from knees to feet, rendered it very painful to walk or stand. Pain in back, in lumbar region and dizziness. He could yet attend his usual avocations. Urinalysis showed nothing unusual concerning the kidneys. After this history and a quick examination I put patient on the anti-syphilitic treatment, and advised rubber bandaging of both lower limbs for the Edema. He improved rapidly under this treatment and gained weight, and I heard nothing more from him (though seeing him every day at his vocation) from the 3d of November, 1901, until January 26, this year. On the last day mentioned patient came into my office hurriedly and asked for something for headache. He had been troubled with it for six weeks and could get no relief. I put him through a brisk purge of mercury, mild chlor., in 10-grain doses, and on some improvement put him on the iodide, and until January 30th he was somewhat relieved, though at all times the headache was persistent. (I will add he had not consulted me concerning his severe headache before 26th January.) He had used patent medicines of different kinds without effect, and the continued severity of his headache drove him to seek other means of relief, when, on the 30th of January, he came to the door of my office and asked for something more for his head. He did not have time to come in. Then, pressing both hands to his head, added: "I am afraid that old disease is going to kill me this time, this head trouble is so persistent." I gave him 15-grain doses of trional at this time, alternately with the iodide.

On Saturday afternoon, February 1, 1902, I was called at his home, where he had staid since I had seen him last, January 30th. While extremely despondent, I suspected nothing unusual mentally. I talked with him something like an hour, and he, in a very despondent way, rehearsed his financial affairs from early manhood. (I will add here he is an extremely industrious man, and is in good circumstances; no cause for financial despondency.) He said he loved money and liked to make it.

After giving him a hypodemic of morphia, ¼ grain, I left him more cheerful, telling him I would call again the next morning. Before I could make my promised call a message came early from the family to come quick—they could not get him to speak. I

suspected contraryness the cause of the alarm, as he was of that disposition; but on examination I was surprised to find dementia. His pulse beat 90, respiration 19, temperature in axilla 101. He was sitting, dressed, in a large rocker, elbows on knees and face buried in hands, and would notice no one. Reflex was good, right pupil responded nicely, left was contracted, had perfect control of all moter muscles and the aphasia was actual. I will add here, during the remainder of his illness I never found his temperature over 99; respiration normal, pulse ranged between 65 and 72.

Fortunately he would take medicine at this time, though insisting upon measuring it himself when permitted to do so. He was as likely to fill the graduate and drink the contents of the bottle as he was the contents of the graduate, when in either case only a drachm was the intended dose. His sight and hearing were not interfered with, and at no time was he unable to go around the room.

My treatment at this time was large doses of the iodide, $\frac{3}{4}$ ounces per diem, with draughts of Vichi water; mercurial inunctions and proto-iodide of mercury almost to ptyalism. I pushed the iodide of potash to tolerance, and gave 1-40 grain strychina alternately. I watched patient with much interest and anxiety, as it was an unusual case to me.

On the morning of the 3rd I hastened my visit, and found he would answer some of my questions by bowing or shaking his head. This much accomplished was quite satisfactory. But I soon learned that he did not appreciate the meaning of my questions. On asking a question, he would answer by bowing; I would then almost instantly repeat the question, and he would shake his head.

He was now undergoing rapid emaciation, and was suffering with meningeal anemia. Iron tonics were advised and continued.

All this treatment was taken and borne well until the tenth day, when patient began improvement. At this time I had to contend with insomnia. He seemed to have a morbid dread of the bed, sat almost constantly in his chair, and persisted in being alone, and when humored would see that all doors were fastened, and when keys were left on inside would lock himself in, and then a general search would go on. He would walk around his bed feeling as far as his arms would reach between the bedding. At this stage he could speak haltingly yes and no, possibly start a sentence but never finishing it, as if he would forget how to speak it; seemed extremely anxious to say something. He would stand before the mantel and measure the board with hands and say ten, and repeat until some one would repeat after him ten, then he would say yes, and sit down, seemingly satisfied.

I tried him on writing. He took the pencil and paper as if he had perfect control of his will, and made one letter, and continued the same letter across the page, then would not attempt further. He could copy a word I might write—for instance, his name.

In the meantime the first part of the day I found him quiet and seemingly reasonable; in the afternoons he became restless and somewhat violent, would resist any restrictions I saw proper to place over him, and positively refused medicine of any description for eight days.

On the 18th of February, at the expiration of the eighth day, he had paced the floor the entire day with pain over the liver. He tried to talk, and made motions to send for me, and after so long made them understand. I found him badly constipated, and soon relieved him of his pain, and from then on I had no difficulty in getting him to take his medicine promptly, and gradual improvement continued.

I will add that his appetite was good throughout his illness. His urine from the third day of his dementia until the fourteenth was scanty and heavily loaded, yet an examination showed nothing unusual.

He is now wonderfully improved, goes to his place of business, but does not attempt to attend to any; waits upon himself, takes his own medicine, etc., but cannot talk, except in that halted manner, and then only a few words audible. He cannot comprehend, except a few simple words spoken plainly.

My object for the report of this important case before this noble assembly, is principally for more information on such cases from more experienced minds.

